# The feasibility of a new self-guided pedicle tap for pedicle screw placement: an anatomical study

**DOI:** 10.1186/s12891-023-06681-7

**Published:** 2023-07-08

**Authors:** Yongtao Liu, Xiaoji Zhou, Yuan Li, Peng Wang

**Affiliations:** 1 Department of Spine Surgery, Affiliated Hospital of Xuzhou Medical University, Xuzhou Jiangsu, 221000 China; 2 Department of Orthopedics, The People’s Hospital of Huishan, Wuxi Jiangsu, 214000 China; 3 Medical Imaging Department, Affiliated Hospital of Xuzhou Medical University, Xuzhou Jiangsu, 221000 China; 4 Department of Clinical Laboratory, Xuzhou Central Hospital, Xuzhou Jiangsu, 221000 China

**Keywords:** Pedicle screw placement, Anatomical study, Tap, Thoracolumbar spine

## Abstract

**Purpose:**

To investigate the safety and accuracy of applying a new self-guided pedicle tap to assist pedicle screw placement.

**Methods:**

A new self-guided pedicle tap was developed based on the anatomical and biomechanical characteristics of the pedicle. Eight adult spine specimens, four males and four females, were selected and tapped on the left and right sides of each pair of T1-L5 segments using conventional taps (control group) and new self-guided pedicle taps (experimental group), respectively, and pedicle screws were inserted. The screw placement time of the two groups were recorded and compared using a stopwatch. The safety and accuracy of screw placement were observed by CT scanning of the spine specimens and their imaging results were graded according to the Heary grading criteria.

**Results:**

Screw placement time of the experimental group were (5. 73 ± 1. 18) min in thoracic vertebrae and (5. 09 ± 1. 31) min in lumbar vertebrae respectively. Screw placement time of the control group were respectively (6. 02 ± 1. 54) min in thoracic vertebrae and (5.51 ± 1.42) min in lumbar vertebrae. The difference between the two groups was not statistically significant (*P* > 0. 05). The Heary grading of pedicle screws showed 112 (82.35%) Heary grade I screws and 126 (92.65%) Heary grade I + II screws in the experimental group, while 96 (70.59%) Heary grade I screws and 112 (82.35%) Heary grade I + II screws in the control group.The difference between the two groups was statistically significant (*P* < 0.05).

**Conclusion:**

The new self-guided pedicle tap can safely and accurately place thoracic and lumbar pedicle screws with low-cost and convenient procedure,which indicates a good clinical application value.

As a symbol of the rapid development of modern spine surgery, the pedicle screw internal fixation technique has been widely used in various spinal disorders, such as trauma, deformity, tumor, and degenerative diseases. In recent years, many scholars have begun to study the use of neurophysiological monitoring, computer navigation and 3D printed guides to achieve accurate placement of pedicle screws, however, which were not widely used in primary hospitals, because of the expensive equipments, prolonged operation time and training of professionals [1–3]. At present, the placement of thoracolumbar pedicle screws is mostly based on the free-hand technique, and the pedicle screws are placed by skill and experiences of the surgeons, which can easily lead to complications such as failure of pedicle screw placement and vascular and nerve injury [4]. Therefore, the author developed a new self-guided pedicle tap (china patent number: ZL201821580271. 9) to improve the safety and accuracy of pedicle screw placement and to study the feasibility of its clinical application through anatomical experiments.

## Materials and methods

### Anatomical material

Eight formalin-fixed and anatomically intact adult spine specimens (T1-L5), four males and four females, excluding trauma, congenital developmental malformations, and scoliosis deformities, were provided by the Department of Anatomy and Research, Xuzhou Medical University. The posterior median incision was taken in the thoracolumbar spine, and the skin, subcutaneous tissue, and lumbar dorsal fascia were incised. The paravertebral muscles were peeled away to expose the vertebral plates, articular processes, and transverse processes. The same spine surgeon placed all pedicle screws in eight spine specimens from T1to L5 on the right and left side of the pedicle, and randomly selected one side of the pedicle to be tapped with a self-guided tap (experimental group) and the other side to be tapped with a conventional tap (control group), in order to compare the indicators related to pedicle screw placement on both sides. The anatomical features including minimum diameter and length of the pedicle channel were observed by radiological measurement, and the difference between the two groups was not statistically significant(*P* > 0.05) (Table 1). All vertebral bodies were measured by peripheral quantitative CT(pQCT) for bone mineral density (Table 2).

**Table 1:** Comparison of anatomical features of spine specimens between two groups

Group	Minimum diameter of pedicle ($\overline{x}$ ± s, mm)		Length of pedicle channel ($\overline{x}$ ± s, mm)	
	Thoracic vertebra	Lumbar vertebra	Thoracic vertebra	Lumbar vertebra
Experimental group	5.62 ± 1.34	8.54 ± 1.73	38.11 ± 1.60	45.85 ± 2.17
Control group	5.64 ± 1.28	8.57 ± 1.06	38.28 ± 1.15	46.52 ± 2.85
*t*	0.1057	0.0935	0.8453	1.1830
*P*	0.9159	0.9257	0.3990	0.2404

Minimum pedicle diameter was the minimum of both transverse and sagittal diameters of pedicles.Length of pedicle channel was the distance from midpoint of posterior pedicle cortex to anterior vertebral cortex along the pedicle axis

**Table 2:** Vertebral bodies bone mineral density

Vertebral body	Bone mineral density ($\overline{x}$ ± *s,*mg/cm^3^)
T1	163.32 ± 32.08
T2	166.39 ± 33.53
T3	167.71 ± 27.05
T4	163.64 ± 31.06
T5	167.79 ± 28.37
T6	165.39 ± 34.56
T7	163.99 ± 29.35
T8	166.38 ± 32.75
T9	164.80 ± 27.96
T10	166.61 ± 34.27
T11	165.16 ± 29.54
T12	167.48 ± 32.34
L1	163.44 ± 32.09
L2	168.03 ± 33.81
L3	163.73 ± 35.03
L4	163.84 ± 29.88
L5	168.31 ± 27.91

### Development of the self-guided taps

**Fig. 1:**
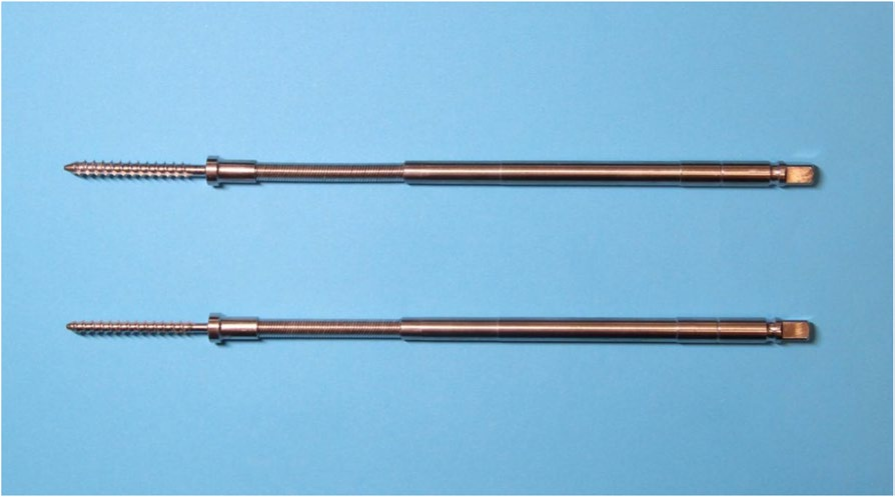
Product of self-guided pedicle tap(overall view)

**Fig. 2:**
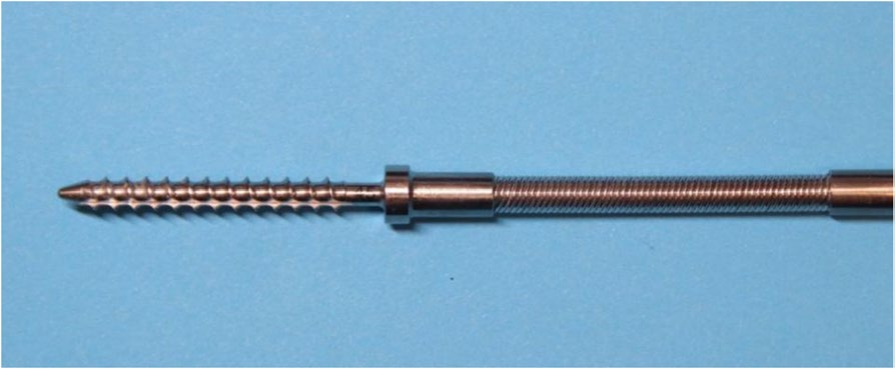
Product of self-guided pedicle tap (details of the threaded end and elastic structure)

**Fig. 3:**
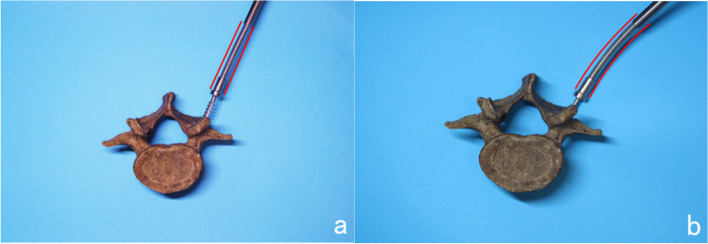
The operation principle of self-guided pedicle taps

The self-guided tap was made of medical stainless steel and consists of a threaded end, elastic structure and handle end (Fig. 1). The tip of the threaded end is designed with a blunt spherical structure to reduce the penetration rate of the tap via pedicles. The threaded end (diameter 4.0–5.5 mm, length 40 mm) can satisfy most thoracic and lumbar pedicle screws placement requirements. A section of elastic structure is provided at the distal end of the threaded end (Fig. 2), which includes an elastic steel cable inside and is surrounded by a spring in the outer layer. The elastic structure can maintain a straight shape when it is not pressed from lateral stress (Fig. 3). It can also bend to the opposite side of the lateral pressure from the pedicle (Fig. 3).

### Method of pedicle screw placement

**Fig. 4:**
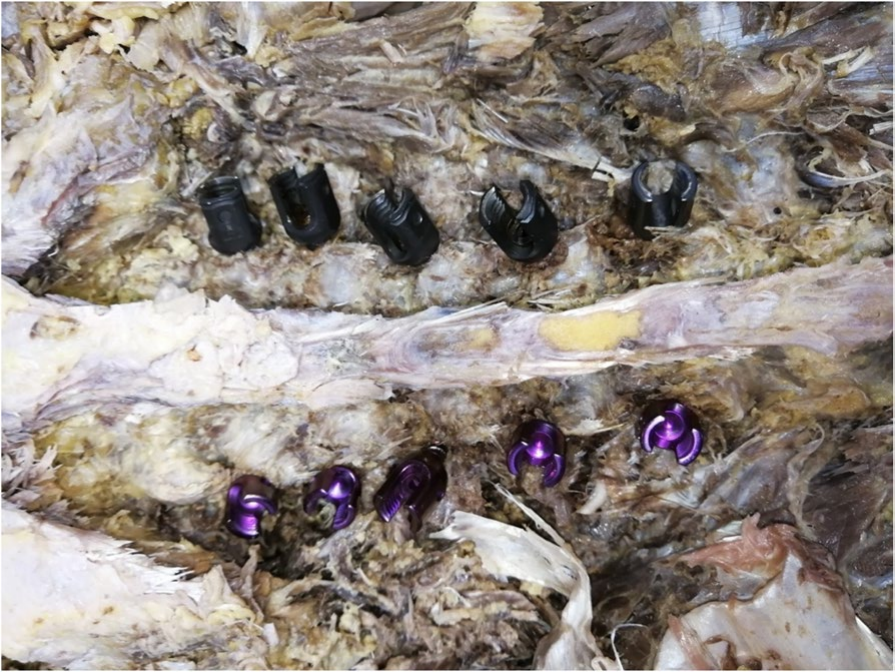
Lumbar pedicle screws placement in cadavers

The method of Fennell [5] was chosen for thoracic screw placement (approximately 3 mm caudal to the lateral border of the transverse and superior articular processes), and the herringbone apex method was chosen for lumbar screw placement (the intersection of the isthmus crest with the accessory process crest). Self-guided tap group (experimental group): The bone cortex was removed at the pedicle screw entry point with a rongeur. The pedicles were drilled with a pedicle opener, and then the new self-guided tap was used to cannulate the pedicles. When the cannulating direction is correct, the elastic structure of the tap maintains the axial direction of the pedicle (Fig. 3a), and the preparation of the pedicle screw channel can be accurately completed.) When the cannulating direction is deviated, the cortical bone of the pedicle outer layer will press the thread end, then the elastic structure could bend to the opposite side (Fig. 3b), correcting the cannulating direction and also the screw channel. After confirming the integrity of the screw channel with a probe, the final screws were cannulated along the prepared screw channel. Conventional tap group (control group): After the opening of the screw entry point, the hole was drilled with an pedicle opener and the integrity of the screw channel was tested with a probe, followed by cannulating of the pedicle with a conventional tap. The screw channel was probed again and then the screws were finally screwed in the pedicles (Fig. 4).

### Observation index

A stopwatch was used to record the time of each screw placement, starting from the removal of the cortical opening at the pedicle entry site and ending when the screw was completely screwed in. The thoracolumbar specimens were scanned with a CT machine (GE Light Speed 16, General Motors, USA) after screw placement. The pedicle screw placement was evaluated by Heary grading criteria: Grade I, entirely contained within pedicle; Grade II, violates lateral pedicle but screw tip entirely contained within the vertebral body (VB); Grade III, tip penetrates anterior or lateral VB; Grade IV, breaches medial or inferior pedicle; Grade V, violates pedicle or VB and endangers spinal cord, nerve root, or great vessels and requires immediate revision [6]. Grade I was defined as accurate pedicle screw placement, and Grade I and Grade II were considered as safe pedicle screw placement.

### Statistical treatments

SPSS software was used for statistical analysis, and quantitative data were expressed as mean ± standard deviation ($\overline{x}$ ± *s*), and independent samples t-test was used for comparison between two groups. Qualitative data were compared using the*χ*^*2*^ analyses, and differences were considered statistically significant (*P* < 0. 05).

## Results

A total of 272 pedicle screws were placed in eight spinal specimens, including 136 screws (4.0–6.5 mm diameter; 35.0–45.0 mm length)respectively in both groups.

### Comparison of screw placement time between two groups

There were no statistically significant differences in screw placement time between the two groups in the thoracic and lumbar segments (Table 3). No additional operation time will be added by the new self-guided tap compared with the conventional tap.

**Table 3:** Comparison of the screw placement time between two groups

Group	Thoracic vertebra		Lumbar vertebra	
	No. of screws	screw placement time ($\overline{x}$ ± s, min)	No. ofscrews	screw placement time ($\overline{x}$ ± s, min)
Experimental group	96	5.73 ± 1.18	40	5.09 ± 1.31
Control group	96	6.02 ± 1.54	40	5.51 ± 1.42
*t*	-	1.4646	-	1.3749
*P*	-	0.1447	-	0.1731

### Imaging measurements of pedicle screw placement

**Fig. 5:**
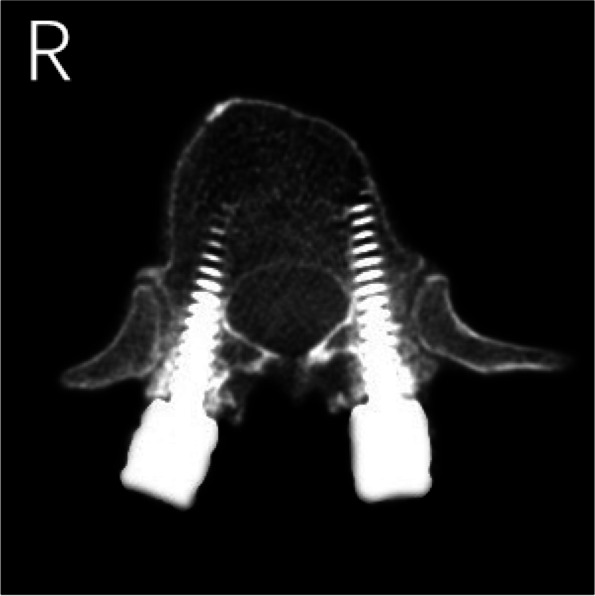
Heary grade I: both screws completely placed in the vertebral pedicle and body

**Fig. 6:**
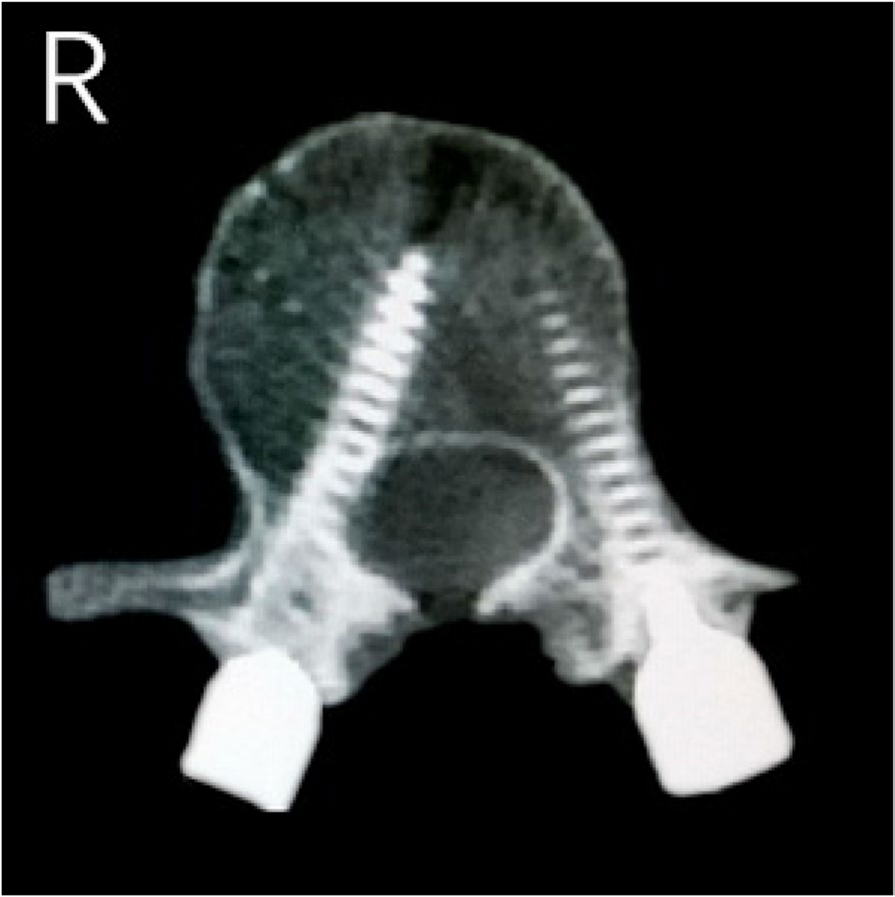
Heary grade II: medial pedicle violation by right screw but screw tip entirely contained within the vertebral body

**Fig. 7:**
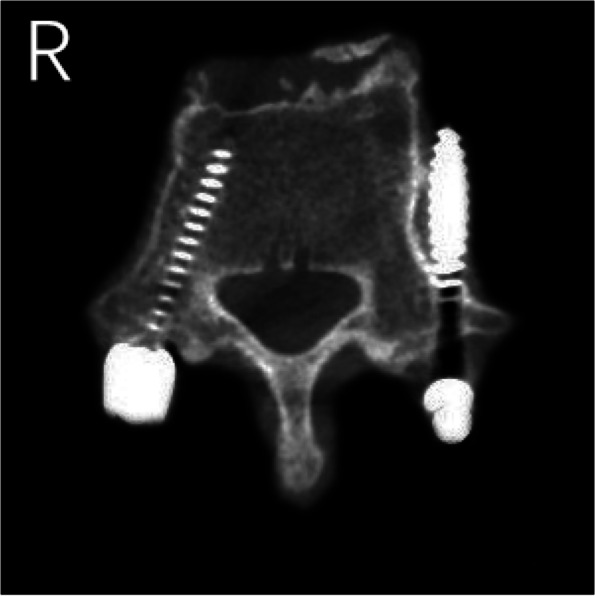
Heary grade III: lateral cortical perforation by left screw

**Fig. 8:**
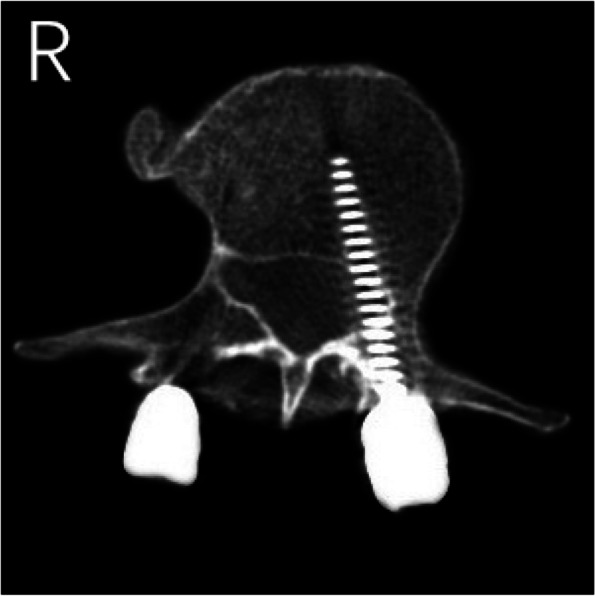
Heary grade IV: medial cortical perforation by left screw

**Fig. 9:**
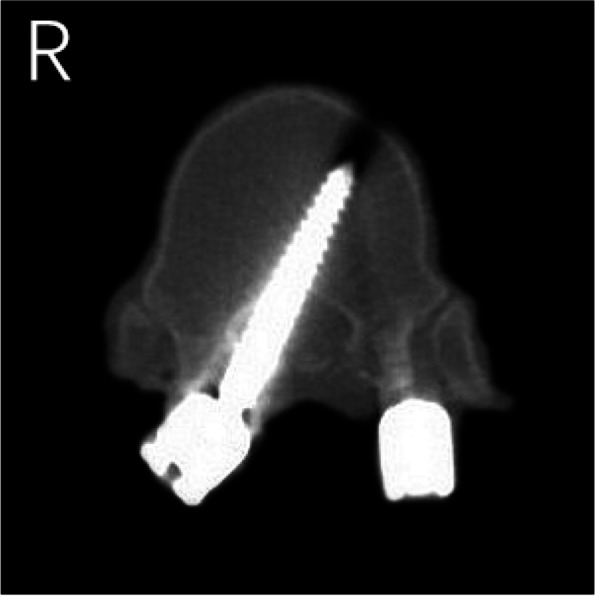
Heary grade V: medial cortical perforation with spinal cord injury by right screw

The results of CT scans after pedicle screw placement were shown in Figs. 5, 6, 7, 8 and 9. The evaluation of pedicle screw placement between the two groups was based on the Heary grading criteria. The rating process of the screws was performed by the same experienced radiologist to ensure the rater reliability and consistency. The statistical results were shown in Table 4. Heary grade I represents the accurate placement of the screw (accuracy), and Heary grade I + II represents the safe screw position (safety). There was a statistically significant difference between the two groups. The accuracy and safety of the experimental group were significantly better than those of the control group.

**Table 4:** Comparison of pedicle screws placement between two groups

Heary grade	No. of Screws(%)		*X*^*2*^	*P*
	Experimental group	Control group		
I	112 (82.35%)	96 (70.59%)	5.2308	0.0222
II	14 (10.29)	16 (11.76%)	0.1499	0.6986
III	6 (4.41%)	12 (8.82%)	2.1417	0.1433
IV	3 (2.21%)	8 (5.88%)	2.3685	0.1238
V	1 (0.74%)	4 (2.94%)	0.8150	0.3666
I+ II	126 (92.65%)	112 (82.35%)	6.5882	0.0103

## Discussion

### The difficulties and risks of thoracolumbar pedicle screw placement

The main difficulties of thoracic pedicle screw placement are as follows: (1) the transverse diameter of the thoracic pedicle is significantly narrow, especially in the upper and middle thoracic segments. The average width of the pedicles in the T4-T8 segments is smaller than 5 mm, so the screws could easily penetrate the pedicle cortex when improperly placed [7]. Helm et al. described a total of 279 thoracic pedicle screws placement, and postoperative CT scans revealed a 14% penetration rate of the medial wall and 68% penetration rate of the lateral wall of the pedicle [8]. (2) The cortex of the thoracic pedicle is closely adjacent to the dura mater, and no reserved space was observed between them. It may cause nerve injury if the medial cortical perforation is more than 2 mm. Therefore, the cortical perforation of the pedicle screw could easily injure the surrounding spinal cord or nerve roots [9]. (3) The thoracic vertebrae are surrounded by many important tissues and organs, such as the lungs and aorta, and poor screw placement may also cause serious complications of these organs such as pneumothorax or aortoclasia. (4) The thoracic vertebrae includes many different segments, representing different anatomical characteristics. Kim et al. [10] noted that the ideal entry site for the thoracic pedicle varies in different thoracic segments, making pedicle screw placement more difficult.

Degenerative diseases of the lumbar spine tend to be more common than the thoracic spine because of the extra stress and mobility. Lumbar spinal stenosis, spondylolisthesis, and degenerative scoliosis often lead to vertebrae rotation, pedicle deformity, and hyperplasia of the facet joints, which could influence the lumbar pedicle screw placement. Therefore, the technique of pedicle screw placement remains an urgent challenge for spine surgery.

### Disadvantages of the traditional free-hand pedicle screw placement technique

The key to free-hand technique are the identification of the entry site and the direction of the screw, which determine the final screw placement. Therefore, enough surgical experience and correct subjective judgment of the screw placement are essential. During the screw placement, surgeons drill into the pedicle with a tap in the direction planned before surgery and adjust the direction according to the resistance from the pedicle. The force and direction are subjectively controlled by hands, and intraoperative postural variations or degeneration of the articular and transverse processes can both influence the screw entry site and screw placement direction. If there is a deviation in the direction or an incorrect entry site, the stainless-steel tap can easily penetrate the pedicle in the process of cannulation, leading to screw loosening, screw placement failure or nerve injury. Complications associated with thoracic pedicle screws by free-hand technique have been reported to be 5.1% to 31.0% [11]. This shows that the reliability and reproducibility of free-hand technique are unsatisfactory. In this study, the screw placement accurate rate(Heary grade I) in the control group was 70.59%, meaning that about 30% of the screws showed different degrees of pedicle penetrations, which further demonstrates the defects of free-hand technique using traditional tools.

### Advantages and disadvantages of the new self-guided pedicle tap

Cagli et al. [12] reported that the thickness of cancellous bone within the pedicle is approximately 1.8 times that of cortical bone. The cortical bone is not as thick as cancellous bone but it shows superior bone density and mechanical strength. The design of the new self-guided tap takes full advantage of this anatomical and mechanical characteristics. In this study, Heary grade I (82.35%) and Heary grade I + II (92.65%) in the experimental group were better than those(70.59% and 82.35% respectively) in the control group. The main reasons were as follows: Based on the above-mentioned mechanical differences between cortical and cancellous bone, the new tap in the experimental group and realizes the self-guiding function via the design of blunt spherical thread tip and elastic structure. When the operator cannulates in the wrong direction, the tip of the tap encounters the cortical bone of the pedicle, which inevitably generates lateral stress, bends the elastic structure and turns the tap toward the center of the pedicle, correcting the operator's misjudgement of the sagittal and horizontal directions. This could reduce the possibility of pedicle penetration and avoid the drawbacks of traditional tap in cannulating. In addition, the results of this study showed no significant difference in screw placement time between both groups, indicating that new tap is simple to learn and easy to operate. The results of this study demonstrate that the new self-guided tap can improve the safety and accuracy of screw placement without prolonging the time of screw placement and it is feasible for clinical application.

It should be noted that the manipulation of new tap is different from the traditional tap in some aspects. For example, with the rigid traditional tap, surgeons could alter directions in tapping process either cranially or caudally or medially or laterally according to the intraoperative fluoroscopy. However, with the new tap this maneuver is not possible because of its elastic structure.

Although the new tap has a certain self-guiding function, it is still necessary to complete the imaging examination and preoperative planning before operation and verify the screw channel before placement. This new tool and technique are not suitable for pedicle fractures or dysplasias. Severe osteoporosis, especially in the cortical bone, can also affect the accuracy of new tap. Moreover, this study was performed on formalin-fixed spinal specimens, the bone quality of which inevitably differ from normal vertebrae in clinical procedures. The samples in this study are relatively few,so further studies on more fresh frozen cadavers or clinical applications are necessary in the future.

In summary, the new self-guided pedicle tap provides a new auxiliary method for the thoracolumbar pedicle screw placement with higher safety and accuracy compared to conventional taps. It is more convenient and economical compared with expensive methods with long learning curve such as computer navigation, 3D-printed navigation templates, or neurophysiological monitoring, promising a wide prospect of clinical application especially in primary hospitals.

## Data Availability

The datasets used and/or analysed during the current study are available from the corresponding author or co-author on reasonable request.
